# Reduced arterial elasticity due to surgical skeletonization is ameliorated by abluminal PEG hydrogel

**DOI:** 10.1002/btm2.10060

**Published:** 2017-05-30

**Authors:** Karyn G. Robinson, Rebecca A. Scott, Anne M. Hesek, Edward J. Woodford, Wafa Amir, Thomas A. Planchon, Kristi L. Kiick, Robert E. Akins

**Affiliations:** ^1^ Nemours ‐ Alfred I. duPont Hospital for Children Wilmington DE 19803; ^2^ Dept. of Materials Science & Engineering University of Delaware Newark DE 19716; ^3^ Dept. of Physics and Engineering, Optical Science Center for Applied Research Delaware State University Dover DE 19901; ^4^ Dept. of Biomedical Engineering University of Delaware Newark DE 19716

**Keywords:** compounds/materials, hydrogel, patient‐targeted therapies, regenerative medicine

## Abstract

Arteries for bypass grafting are harvested either with neighboring tissue attached or as skeletonized vessels that are free of surrounding tissue. There are significant benefits to skeletonization, but reports suggest that skeletonized vessels may develop structural defects and are at risk for atherosclerosis. We investigated the specific short‐term effects of skeletonization on carotid artery biomechanics and microanatomy in a rabbit model. Six carotid arteries were surgically skeletonized. To support healing, three of these received polyethylene glycol hydrogel injected along their exterior surfaces. M‐mode ultrasonography was used to track circumferential cyclic strain in the skeletonized, hydrogel‐treated, and contralateral vessels. On day 21, the arteries were harvested, and vessel structure was assessed by histology, immunofluorescence microscopy, two‐photon elastin autofluorescence, and second harmonic generation (SHG) microscopy. Intimal‐medial thickness appeared unaffected by skeletonization, but the SHG signals indicated significant changes in collagen turnover in the adventitia. Skeletonized arteries also exhibited significantly decreased radial compliance (circumferential cyclic strain dropped ∼30%) and decreased numbers of elastic laminae (9.1 ± 2.0 to 2.3 ± 1.4). Hydrogel treatment protected against these effects with treated vessels maintaining normal mechanical properties. These results indicate that arterial skeletonization triggers immediate effects on vessel remodeling and reduced vessel compliance resulting in specific tissue alterations within 21 days, but that these effects can be attenuated by the placement of hydrogel on the exterior surface of the skeletonized vessel.

## 1 INTRODUCTION

Surgical grafting and reconstruction are principal treatments for coronary and peripheral artery diseases^1^ and for the repair of congenital cardiovascular malformations.^2^ Two established harvesting techniques, pedicled and skeletonized, are used for arterial grafts. In the preparation of pedicled grafts, the artery is dissected away from the surrounding tissue with the accompanying vasa vasorum, fascia, adipose tissue, and lymphatics intact, while skeletonization requires the artery to be dissected free of all surrounding tissue.^3^ In the case of coronary artery bypass grafting using internal thoracic arteries (ITAs), the benefits of skeletonized harvesting of graft tissue include increased graft flow,^4^ increased graft length,^5^, ^6^ decreased incidence of mediastinitis,^5^ and reduction in postoperative pain.^7^, ^8^, ^9^ For these reasons, skeletonization has increased in popularity and has become the preferred method of many surgeons. There are, however, a number of risks associated with this method of harvesting.

Arterial skeletonization may adversely affect graft structure and long‐term resistance to atherosclerosis,^10^ and if an artery is partially denuded of adventitium and its vasa vasorum during skeletonization, areas of degeneration may develop.^10^ Studies show significant negative effects of separating an artery from its surrounding tissue. Mechanical injury during harvesting predisposes the artery to spasms,^11^ which can cause graft occlusion, intimal hyperplasia, and early graft failure.^10^, ^12^, ^13^ One study found degenerative changes in skeletonized ITAs and radial arteries compared to pedicled ITAs and RAs, including: splitting of internal elastic laminae, reduced tortuosity of the internal elastic laminae, and thickening and detachment of the intima.^11^ Others have shown that experimental interruption of the aortic vasa vasorum led to abnormal morphology of elastin and collagen fibers of the outer media, which resulted in arterial wall stiffening within 15 days.^14^ In a canine model, skeletonized ITAs stripped of their adventitia had a higher incidence of thrombosis, intimal thickening, and medial injury than did pedicled and free ITA grafts.^15^ Thus, although ITA skeletonization allows increasing the graft's length and decreasing sternal ischemia, invisible macroscopic damage to the artery wall may precipitate remodeling.^16^ Mechanical damage associated with skeletonization may also affect vessel structure with the possibility of stenosis, fibrosis, and vessel failure, which are significant problems driven by maladaptive cellular responses elicited by tissue injury and hemodynamic stress.^17^

To understand better the effects of skeletonization on arteries and potentially to ameliorate some of the negative effects associated with skeletonization, we investigated the placement of a polyethylene glycol (PEG) hydrogel with thiol‐maleimide mediated click chemistry on the abluminal surface of skeletonized arteries. PEG was selected because it has been widely used and characterized as a biomaterial for tissue engineering applications, including wound healing.^18^, ^19^, ^20^, ^21^, ^22^, ^23^, ^24^, ^25^, ^26^ PEG polymers have low toxicity and are approved by the Food and Drug Administration for clinical use in numerous contexts.^27^ We have previously reported strategies for manipulating gelation and degradation of PEG‐based hydrogels that are injectable and biocompatible.^28^, ^29^, ^30^, ^31^, ^32^ These gels can be formed rapidly^28^ and simply for controlled administration in our experimental surgery setting. The current study was undertaken to investigate biomechanical, cellular, and extracellular changes that result from artery skeletonization with or without placement of hydrogel on the abluminal surface of the vessel.

## 2 MATERIALS AND METHODS

### 2.1 Animal care and surgery

Following Institutional Animal Care and Use Committee approval, carotid artery skeletonization was assessed using New Zealand White rabbits (Covance, Princeton, NJ). Perioperatively, animals received analgesia (0.3 mg/kg meloxicam, Norbrook Laboratories, Northern Ireland) and prophylactic anitbiotic (50 mg/kg cefazolin, Hospira Inc., Lake Forest, IL). Animals were anesthetized for surgery with intramuscular ketamine (35 mg/kg, Mylan Institutional LLC, Rockford, IL) and xylazine (5 mg/kg, Lloyd Laboratories, Shenandoah, IA), and placed on a warming blanket during surgery. All procedures were carried out using aseptic technique. An approximately 4 cm incision was made, and the underlying tissue was dissected to expose the major blood vessels on the left side of the neck. The left common carotid artery was skeletonized (i.e., freed from the surrounding tissue). For animals in the hydrogel group (*n* = 3), 0.5 ml of PEG hydrogel was introduced along the skeletonized region of the artery using a double‐barrel syringe. The incision was approximated with 5‐0 Vicryl sutures (Ethicon, Somerville, NJ).

### 2.2 Ultrasound imaging and analysis

Ultrasonography was performed on the left and right common carotid arteries of all animals one day prior to surgery, immediately following surgery, and 3, 7, 14, and 21 days following surgery using a Vevo 2100 (VisualSonics, Toronto, Ontario) with a 40 MHz MicroScan transducer. High‐resolution, two‐dimensional cine loops in brightness mode (B‐mode; 300 frames) and motion mode (M‐mode; 3‐5 second acquisition) were acquired in transverse view. Circumferential cyclic strain was calculated from M‐mode luminal diameter measurements to determine maximum and minimum vessel wall displacements corresponding to peak systolic (Ds) and end diastolic pressure (Dd). The Green‐Lagrange circumferential cyclic strain was calculated using: ½ [(Ds/Dd)^2^ – 1] × 100%.

### 2.3 Tissue harvest and histology

Animals were humanely euthanized 21 days after surgery with Euthasol (VedCo, St. Joseph, MO) delivered IV using an ear‐vein catheter to achieve a dose of at least 0.22 ml/kg. The operated and contralateral carotids were harvested, fixed in 4% paraformaldehyde (Electron Microscopy Sciences, Hatfield, PA), dehydrated in graded ethanol, and paraffin‐embedded. Transverse sections (5 µm thick) were deparaffinized, stained with hematoxylin and eosin, and digitally imaged in brightfield using an EVOS FL Imaging System (Life Technologies, Carlsbad, CA). Average intima‐media thickness (IMT) was determined by measuring six regions in each artery using Image Pro Plus software (version 6.3; Media Cybernetics, Rockville, MD). For immunostaining, deparaffinized artery sections were used with antigen retrieval accomplished by steam heating sections in 10 mM citrate buffer pH 6.0 for 30 min. Samples were then stained with anti‐α smooth muscle actin (αSMA, Abcam, clone 1A4, 1:100 dilution in PBST) followed by an Alexa Fluor 488 conjugated secondary antibody and Hoechst 33258. Samples were digitally imaged on an Olympus BX‐60 fluorescence microscope equipped with a Retiga 6000 14‐bit digital camera (QImaging, Surrey, British Columbia) controlled by Image Pro Plus software.

### 2.4 Nonlinear optical microscopy

Paraffin‐embedded sections were imaged using a Zeiss LSM 780 confocal microscope with a 3 W Chameleon Vision II (Coherent Inc., Santa Clara, CA) Ti:Sapphire oscillator used to generate 140 fs laser pulses at 820 nm with a 80 MHz repetition rate. A 40× 1.4 numerical aperture (NA) PlanApo oil immersion objective (Zeiss 420762‐9900) and 1.2 NA water immersed condenser (Zeiss, Oberkochen, Germany) were used. The 410 nm, second harmonic signals generated in the tissue were detected using a 400 ± 20 nm filter (Chroma Technology Corp., Bellows Falls, VT), and a 525 ± 50 nm filter (Chroma Technology Corp.) was used for two‐photon excited fluorescence (TPEF) detection. TPEF was used to visualize elastin autofluroescence and a nondescanned detection (NDD) system was used for detection of SHG in the forward direction using a photomultiplier tube (LSM NDD; Zeiss) and in the reverse direction using a GaAsP detector (LSM BiG; Zeiss) to visualize collagen.^33^, ^34^

### 2.5 Elastin analysis

The elastic laminae were enumerated in six regions of each artery using acquired digital images.

### 2.6 Collagen analysis

For each arterial cross‐section, Z stacks were acquired across the entire artery, typically 1.7 by 1.7 mm with 16‐bit gray scale resolution. The ratio of the forward‐emitted to backward‐emitted SHG (F/B) was calculated using an analysis procedure based on Burke et al.^35^ In short, a single confocal plane was selected based on local contrast, and three 125 by 125 micron (600 by 600 pixels) Regions of Interest (ROI) were selected. Masks were created by processing each ROI to identify pixels contributing to SHG F/B values with a lower threshold set to remove pixels with intensity below 750 containing only noise, and an upper intensity of 65,000 to avoid saturated pixels. The forward and backward masks were combined to give a final ROI mask that contained only pixels that were present in each mask. The ratio of the forward to the backward SHG images was calculated for each included pixel to create “F/B images” for each ROI. Average F/B values for pixels in the F/B images were used to assess differences between the artery that underwent surgery and its contralateral control. For comparisons between animals, F/B values from the surgically manipulated arteries were normalized to the mean value of the respective control vessel with a normalized F/B of 1 indicating no difference between the control and sample.

### 2.7 Hydrogel preparation

Four‐arm star, maleimide‐functionalized PEG (PEG‐(MAL)_4_, *f* = 4, *M*_n_ 10,000 g/mol, JenKem Technology, Beijing, China) and four‐arm star, thiol‐functionalized PEG (PEG‐(SH)_4_, *f* = 4, *M*_n_ 10,000 g/mol, JenKem Technology) were employed to form gel networks. For in vitro studies, fibronectin (BD Biosciences, Franklin Lakes, NJ), bFGF (Peprotech, Rocky Hill, NJ), and low molecular weight heparin (LMWH; Celsus, Cincinnati, OH) were included for cell attachment, support of cell growth, and growth factor incorporation as described previously.^36^ While these components allow for cell adhesion and proliferation on the surface of the hydrogels, our previous studies confirm that their inclusion in the hydrogels does not affect hydrogel mechanical properties.^36^ Briefly, PEG‐(MAL)_4_ was mixed with fibronectin (100 nM) and PEG‐(SH)_4_ was mixed with maleimide‐functionalized LMWH^36^ (0.1 mM) and bFGF (60 nM) in 10 mM citrate buffer, pH 4.5. After sterile filtration, the PEG solutions were mixed to create 70‐µl hydrogels in 96‐well tissue culture plates that were refrigerated prior use. Three types of hydrogel were prepared by altering the total PEG concentration (3 wt%, 4 wt%, and 8 wt%) with 3 wt% comprising 2 mM PEG‐(MAL)_4_ and 1 mM PEG‐(SH)_4_; 4 wt% hydrogels comprising 2 mM PEG‐(MAL)_4_ and 2 mM PEG‐(SH)_4_; and 8 wt% hydrogels comprising 4 mM PEG‐(MAL)_4_ and 4 mM PEG‐(SH)_4_.

For in vivo studies, 4 wt% hydrogels were prepared as described above except the fibronectin, bFGF, and LMWH were excluded. Each component was dissolved in 10 mM citrate buffer, pH 6.1 and sterilized by 0.2 µm filtration. Subsequently, 250 µL of each solution were mixed using a double‐barrel syringe and mixing tip (MedMix Systems AG, Risch‐Rotkreuz, Switzerland); changes in viscoelasticity were immediately apparent after injection, and gelation was complete within 10 seconds. All components used in vivo passed Limulus Amebocyte Lysate assays (Lonza, Walkersville, MD) prior to implantation.

### 2.8 Cell culture, microscopy, and viability assay

Adventitial fibroblasts were isolated from freshly dissected rabbit aorta that was rinsed and ligated with suture. The external vessel surface was exposed to collagenase type II (1 mg/ml, Worthington Biochemical Corporation, Lakewood, NJ) to liberate adventitial cells. Cells were cultured in Stromal Cell Growth Medium (Lonza) and maintained at 37°C in a 5% CO_2_ incubator. Cells were seeded on the surface of 70‐µl hydrogels in 96‐well plates (0.3 cm^2^ surface area) at a density of 12,500 cells/cm^2^ in 100 µL medium. Live cells were viewed using a Leica Fluovert inverted microscope with Hoffman Modulation Contrast optics using a Hamamatsu ORCA camera (Hamamatsu Photonics, Japan) and Image Pro Plus software. Cell attachment and proliferation were assayed using Cell Titer Blue (CTB, Promega, Madison, WI). To assess attachment, CTB was added to the culture medium 2 hours after seeding and incubated for two additional hours. The medium was analyzed using a VICTOR X4 2030 Multilabel Plate Reader (PerkinElmer, Waltham, MA) at excitation/emission wavelengths of 550/590 nm. Measurements were repeated at 24, 48, and 72 hours in culture, and the change in signal was used to estimate cell number changes over time.

### 2.9 Rheological characterization of hydrogels

Hydrogel mechanical properties were determined via bulk oscillatory rheology.^36^ Briefly, hydrogels were formed directly on an AR‐G2 rheometer (AR‐G2, TA instruments, New Castle, DE) by mixing hydrogel precursor solutions (as given above, at room temperature). A 20‐mm diameter stainless steel cone and plate geometry (1°) was used in all measurements. Time‐sweep measurements were obtained within the linear viscoelastic regime using 1% constant strain and 6 rad/s angular frequency. The storage moduli after reaching the plateau region were recorded as the final storage moduli.

### 2.10 Statistical analyses

SPSS 22 (IBM, North Castle, NY) was used to perform two‐way ANOVA with post hoc Tukey HSD for circumferential strain and IMT measurements, one‐way ANOVA with post hoc Tukey HSD for elastic laminae measurements, and Kruskal–Wallis for F/B ratio. *p* Values of <.05 were considered significant.

## 3 RESULTS

### 3.1 Animal surgery

**Figure 1:**
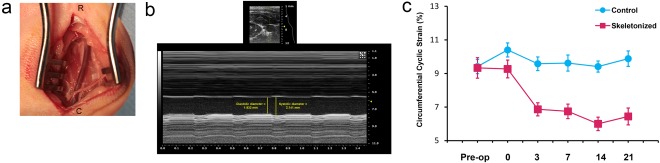
(a) View of the surgical field following skeletonization of the left common carotid artery in a rabbit (R = rostral, C = caudal). (b) Circumferential cyclic strain values calculated from M‐mode diameter measurements. The distance between two lines of strong specular reflection seen in the anterior and posterior vessel walls was used to determine maximum and minimum vessel wall dimensions^37^ corresponding to peak systole and end diastole. To calculate Green‐Lagrange circumferential cyclic strain, the following formula was used^38^: ½ [(Ds/Dd)^2^ – 1] × 100%. (c) Circumferential cyclic strain of unoperated carotid arteries (circles, *n* = 6) and skeletonized carotid arteries (squares, *n* = 3) over 21 days. Error bars represent standard error

In this study, we characterized the effects of skeletonization on the structure and elastic properties of the carotid artery. Three, healthy, male New Zealand White rabbits underwent surgery to skeletonize the left common carotid artery (Figure 1a). All animals were between 5 and 8 months old and weighed from 3.0 to 3.1 kg. All surgeries were performed in the same manner.

### 3.2 Ultrasound and cyclic strain analysis

Animals underwent ultrasound imaging (Figure 1b) to characterize both the right (control) and left (operated) common carotid arteries one day prior to surgery, immediately following surgery, and postoperatively on days 3, 7, 14, and 21. Green‐Lagrange cyclic strain values were calculated from M‐mode diameter measurements (Figure 1c). Preoperatively, the mean cyclic strain was 9.4 ± 1.8% for control vessels (right common carotid arteries, *n* = 6) and 9.3 ± 1.8% for vessels assigned to the skeletonization group (*n* = 3). The mean cyclic strain for control vessels remained consistent throughout the course of the study (10.4 ± 1.8% immediately postoperatively, 9.6 ± 1.7% on day 3, 9.6 ± 2.0 on day 7, 9.4 ± 1.4% on day 14, and 9.9 ± 2.0% on day 21). Cyclic strain values for skeletonized vessels remained at 9.3 ± 1.6% immediately postoperatively, but these vessels exhibited significantly decreased strain compared to controls on days 3 (6.9 ± 1.2%, *p* = .0003), 7 (6.7 ± 1.3%, *p* = .0008), 14 (6.0 ± 1.2%, *p* = .0001), and 21 (6.4 ± 1.5%, *p* = .0001).

### 3.3 Intima‐media thickness analysis

**Figure 2:**
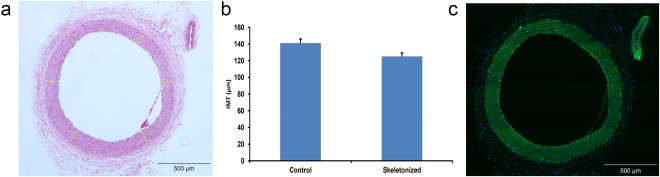
(a) Rabbit common carotid artery in cross section. Tissue was harvested 21 days after surgery and stained with hematoxylin and eosin; IMT distances were measured along six radial directions as indicated. (b) IMT measurements for unoperated carotid arteries (*n* = 6) and skeletonized carotid arteries (*n* = 3). Error bars represent standard error. (c) Immunofluorescence detection of smooth muscle cells using an antibody to αSMA (green) and Hoechst 33258 to visualize nuclei (blue)

Carotid arteries harvested 21 days after surgery were stained with hematoxylin and eosin and analyzed as shown in Figure 2a. IMTs were determined from H&E images by averaging six measurements from each section and are shown in Figure 2b. No significant differences were seen in IMT between control (141.0 ± 29.4 µm) and skeletonized (125.1 ± 18.1 µm) arteries. Sections were deparaffinized and fluorescently stained for αSMA to assess the organization of the media (Figure 2c). αSMA appeared similar in control and skeletonized vessels.

### 3.4 Two‐photon elastin fluorescence and immunofluorescence imaging and analysis

**Figure 3:**
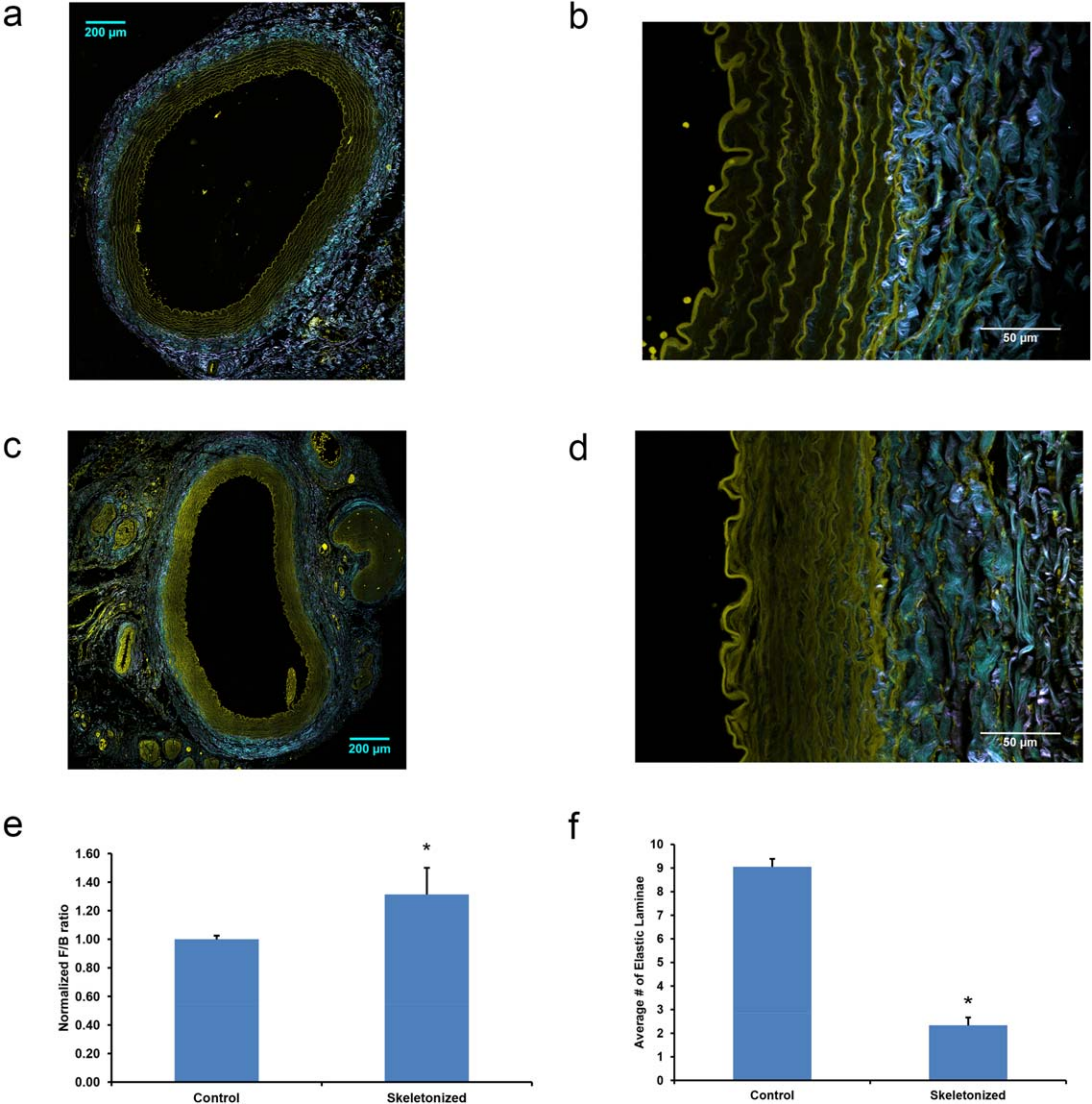
(a) Carotid arteries were harvested 21 days after surgery. Representative image of a control rabbit carotid artery using TPEF (yellow) and SHG in the forward (magenta) and reverse (cyan) direction. (b) Representative image of elastin organization in a control rabbit carotid artery using TPEF (yellow). (c) Representative image of a skeletonized rabbit carotid artery using TPEF (yellow) and SHG in the forward (magenta) and reverse (cyan) direction. (d) Representative image of elastin organization in a skeletonized rabbit carotid artery using TPEF (yellow). (e) The F/B SHG ratios of control (*n* = 6) and skeletonized (*n* = 3) arteries. (f) The number of elastic laminae present in sections of carotid from control (*n* = 6) and skeletonized (*n* = 3) arteries. Error bars represent standard error and * indicates *p* < .05

Paraffin‐embedded transverse sections of the carotid arteries were imaged by TPEF to visualize elastin. Representative TPEF images are shown in Figure 3. In control vessels, elastin was present in concentric fenestrated sheets separated by vSMCs as is typical in large arteries.^39^ However, in skeletonized vessels, elastin appeared to be highly fragmented and the skeletonized vessels had significantly fewer elastic laminae (2.3 ± 1.4) compared to the control (9.1 ± 2.0; *p* < .001) vessels (Figure 3f).

### 3.5 Second harmonic generation imaging and analysis

Paraffin‐embedded transverse sections of carotid arteries were imaged by SHG to visualize the distribution of collagen. Forward and backward scatter SHG signals were collected. Representative images are shown in Figure 3. As expected, collagen was found predominantly in the adventitial layer and appeared predominantly in circumferential layers with areas of local helical arrangement. This general organization is typical of arteries.^33^, ^40^, ^41^ The appearance of collagen did not appear to vary across the samples evaluated; however, the ratio of forward to backward SHG signals (F/B ratio) was, on average, 1.31‐fold higher in skeletonized vessels than control (*p* = .003), suggesting significant alterations in collagen structure in the skeletonized vessels (Figure 3e).

### 3.6 Hydrogel design and characterization

**Figure 4:**
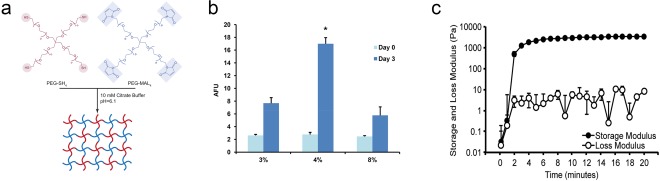
(a) Schematic of PEG‐(MAL)_4_ and PEG‐(SH)_4_ hydrogel formation used in vivo. (b) Cell Titer Blue results for rabbit adventitial fibroblasts cultured on PEG hydrogels. The mean fluorescence is reported as an estimate of the number of viable cells after 2 hours (day 0) and 72 hours (day 3) in culture. Error bars represent standard error (*n* = 3 hydrogels for each wt%) and * indicates *p* < .05. (c) Representative time‐sweep analysis of the gelation of 4 wt% hydrogel via oscillatory rheology; an average final elastic modulus of 3.5 ± 0.5 kPa was observed (*n* = 3)

Hydrogels, including PEG‐based hydrogels,^42^, ^43^ are widely used for wound healing applications, and we investigated the possibility that placement of hydrogel along the exterior of skeletonized arteries would attenuate maladaptive responses. PEG‐based hydrogels were chosen because of their hydrophilicity, biocompatibility, and chemical flexibility. Maleimide‐ and thiol‐functionalized PEG macromers were used to generate stable crosslinks via a Michael‐type addition reaction (Figure 4a). To confirm the biocompatibility of these PEG hydrogels for use in rabbits, and to determine an optimal weight percent gel to employ in the study, adventitial fibroblasts (AFs) isolated from rabbit aorta were seeded on the surface of 3, 4, and 8 wt% hydrogels. The modulus of the hydrogels was altered by varying the total amount of PEG.^36^ To support cell adhesion and growth, these gels contained fibronectin, LMWH, and bFGF at levels that encouraged cell attachment but that did not affect the modulus of the hydrogel network as shown in our previous work.^36^ The cells displayed typical fibroblast morphology with regions of extensively spread cytoplasm on all three moduli. There was no evidence of cell toxicity, consistent with previous results using similar materials.^31^, ^32^, ^36^ The rabbit cells were similar in appearance to human AFs used in our previous work,^36^ and the rabbit AFs did not vary significantly in cell area or ellipticity across the three hydrogel concentrations (data not shown). Cell attachment and proliferation were assessed using a Cell Titer Blue (CTB) assay (Figure 4b). Cells were seeded at the same density on each modulus gel. Two hours after seeding (i.e., Day 0), the cells showed a similar degree of attachment on all gels. By day 3, there was a significant difference in CTB fluorescence on the gels (*p* = .0026, ANOVA) with the 4 wt% hydrogels showing significantly enhanced proliferation compared to 3 wt% (*p* = .0075, Tukey HSD) and 8 wt% (*p* = .003, Tukey HSD). Not surprisingly, the hydrogel formulation that supported maximal rabbit AF proliferation (i.e., 4 wt% PEG) differed from the formulation found to support maximal proliferation in our previous studies of human AFs (i.e., 8 wt% PEG) since the substrates supporting maximal proliferation vary depending on both cell type and species.^36^ Based on the results of our cell culture testing, we selected 4 wt% hydrogels for use in animal studies since this formulation best supported rabbit AF growth in vitro.

The modulus of the 4 wt% PEG gels for the animal studies was determined using oscillatory rheology. The storage (G′) and loss moduli (G″) were measured as a function of time to estimate time to gelation and determine final moduli under in vitro conditions (Figure 4c). The average storage modulus for 4 wt% PEG hydrogels of this type was determined to be 3.5 kPa. Exclusion of LMWH, growth factor, and/or fibronectin from the PEG hydrogels exhibited no measurable changes in gel modulus compared to gels containing these factors (data not shown).^36^

### 3.7 Effect of hydrogel implantation

**Figure 5:**
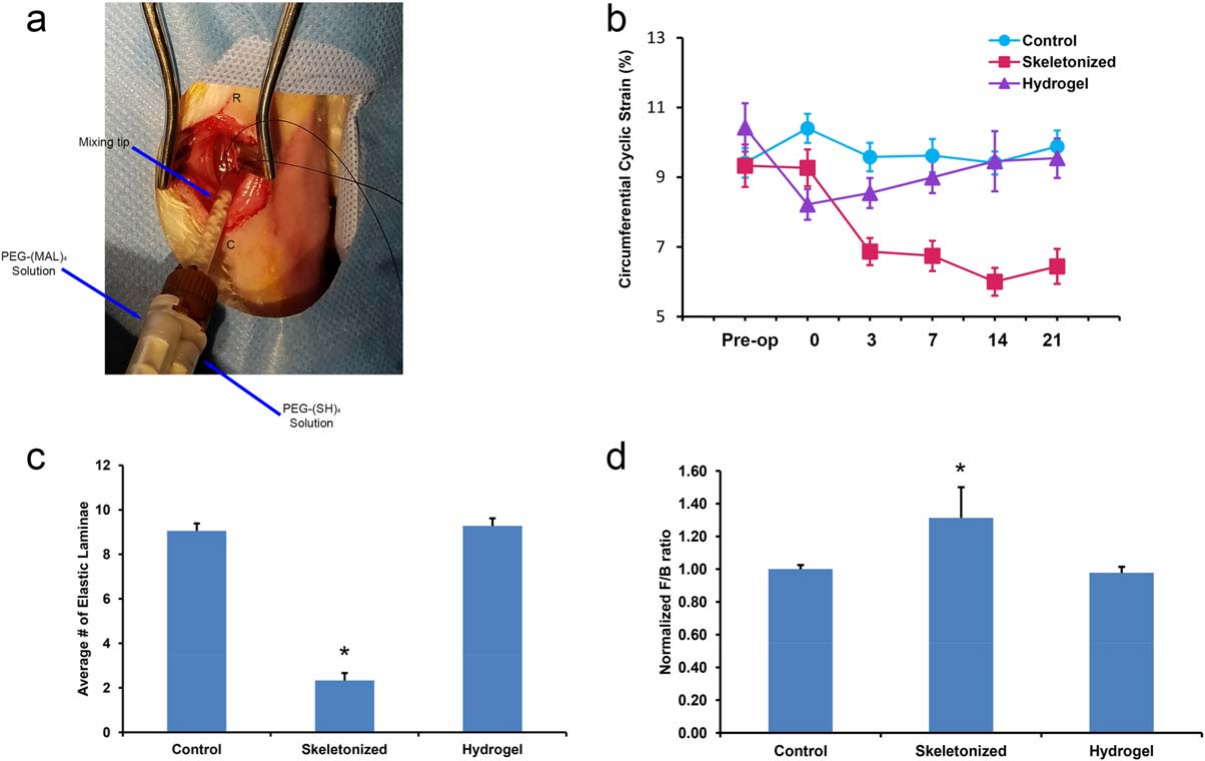
(a) PEG hydrogel placed along the surface of a skeletonized rabbit carotid artery using a double‐barrel syringe with mixing tip (R = rostral, C = caudal). (b) Circumferential cyclic strain of unoperated carotid arteries (circles, *n* = 6), skeletonized carotid arteries (squares, *n* = 3), and skeletonized carotid arteries treated with hydrogel (triangles, *n* = 3) over 21 days. Error bars represent standard error. (c) The number of elastic laminae present in sections of carotid from control (*n* = 6), skeletonized (*n* = 3) arteries, and skeletonized arteries treated with hydrogel (*n* = 3). Error bars represent standard error. (d) The F/B SHG ratios of control (*n* =6), skeletonized (*n* = 3) arteries, and skeletonized arteries treated with hydrogel (*n* = 3). Error bars represent standard error. *p* < .05 are indicated by *

Three, healthy, male New Zealand White rabbits underwent surgery to skeletonize the left common carotid artery. All animals were between 6 and 8 months old and weighed 3.2–3.4 kg. After skeletonization, the animals received 4 wt% hydrogel, which was injected along the vessel surface. All surgeries were performed in the same manner. Gels were formed in situ by combining equimolar quantities of PEG‐(MAL)_4_ and PEG‐(SH)_4_ (Figure 5a). Changes in viscoelasticity were immediately apparent after injection, and gelation was complete in less than 10 seconds as assessed by residual gel on the injector tip. There was no visible dispersion of PEG away from the administration site, indicating rapid and complete crosslinking of the hydrogel and minimal diffusion of precursors from the injection site. Given the significant thiol and antioxidatve capacities of extracellular fluids,^44^ it is likely that any un‐reacted functional groups on the gel surface were quickly neutralized. The intact hydrogel was visible by ultrasonography through 21 days and exhibited no evidence of toxicity or adverse effects.

Preoperatively, the mean cyclic strain was 10.4 ± 2.1% for vessels assigned to the hydrogel group (*n* = 3). Vessels that received hydrogel had an initial drop in cyclic strain (Figure 5b) postoperatively (8.2 ± 1.3%; *p* = .0067 compared to controls), but this was followed by increasing cyclic strain throughout the course of the study back to control levels (8.5 ± 1.3% on day 3, 9.0 ± 1.3% on day 7, 9.5 ± 2.6% on day 14, and 9.5 ± 1.7% on day 21). There were no statistically significant differences in cyclic strain between control vessels and vessels receiving hydrogel from days 3 to 21. IMT values for vessels receiving hydrogel (145.7 ± 35.5 µm) were not significantly different from those of control or skeletonized vessels. Vessels that received hydrogel treatment had intact medial elastin and significantly more elastic laminae (9.3 ± 1.4; Figure 5c) than vessels that were skeletonized alone (*p* < .001). The average normalized F/B SHG ratio was significantly higher in skeletonized vessels (1.31) than in hydrogel‐treated vessels (0.98, *p* = .003), indicating a difference in collagen structure. The F/B SHG ratio was not significantly different between hydrogel treated vessels and unoperated controls (*p* = .495; Figure 5d).

## 4 DISCUSSION

The results presented here indicate that skeletonization of rabbit carotid artery leads to significant biomechanical, matrix, and cellular changes indicative of maladaptive tissue responses beginning within 72 hours of surgery. These negative effects were attenuated by the placement of PEG hydrogel on the abluminal surface of the skeletonized vessel. The skeletonized vessels in this study had altered biomechanical properties exhibiting significantly lower circumferential cyclic strain beginning within 72 hours after surgery. The rapid decrease in strain is sustained over three weeks, suggesting that an early tissue response to skeletonization alters the tissue mechanics and tissue compliance. This reduction in cyclic strain indicates that the skeletonized vessels distended significantly less than the unoperated control vessels under the same changes in systemic pressure. During the cardiac cycle, carotid arteries expand under systolic pressure then elastically recoil during diastole. This diastolic recoil, or Windkessel function, is an important characteristic of large arteries that sustains diastolic pressure further into the arterial arbor and capillaries. The decrease in cyclic strain observed after skeletonization is, thus, a physiologically significant effect, although reduced carotid function in the rabbit would be expected to have little downstream effect due to collateral circulation from the circle of Willis.^45^ Nevertheless, the decreased arterial distensibility is consistent with increased stiffness, decreased elasticity, or both^46^ and is generally associated with vessel wall alterations.

The skeletonized vessels in this study also exhibited disrupted collagen and elastin. The arterial wall combines multiple structurally significant components including elastic laminae, collagen fibers, and smooth muscle cells (SMCs). Together, these constituents determine the fundamental mechanical responses of the arterial wall.^47^ Skeletonized vessels had fragmented elastin and significantly fewer elastic laminae compared to controls. Elastin fibers form a resilient network that contributes to arterial wall elasticity. Interestingly, in addition to critical circulatory and biological impacts,^48^, ^49^ loss of elastin has direct biomechanical consequences on arteries. Disrupted elastin networks are common features of abdominal aortic aneurysm^50^, ^51^, ^52^, ^53^ and are associated with anatomic changes and alterations in vessel wall stiffness.^54^, ^55^, ^56^ Hypertension also results in arterial stiffening and matrix changes including altered collagen/elastin ratios.^33^ In animal models, Fonck et al.^57^ showed that elastin degradation dramatically increases the stiffness of mouse and rabbit carotid arteries, and Phillips et al. found that small amounts of elastase significantly reduced circumferential cyclic strain in rats over 28 days with associated mechanical damage.^37^ Thus, the loss of elastin in our study may account for the decreased strain, although this implies that the elastin was disrupted within 72 hours after surgery.

SMCs in the media are the cells responsible for depositing elastin along with other matrix proteins such as collagen type I, collagen type III, collagen type IV, and fibronectin, which play integral roles in maintaining the mechanical integrity of blood vessels.^58^ Decreased elastin expression in SMCs is indicative of a phenotypic switch from the contractile state to a synthetic phenotype. This switching can be triggered by vessel remodeling, injury, or disease.^59^ Inadequate elastin deposition by synthetic SMCs compromises the mechanical integrity of vessel constructs.^60^ Elastin also affects SMC organization,^61^ and in elastin knockout (Eln–/–) mice, aortic SMCs lose their circumferential orientation and cell‐cell contacts and over‐proliferate, leading to occlusion of the vessel lumen.^62^ While we did not find evidence of intimal hyperplasia or changes in SMC organization, it is possible that changes would be evident later than 21 days after surgery.

The roles of elastin and collagen in arterial mechanics are thought to be highly coupled and interrelated,^57^, ^63^, ^64^, ^65^ and the combination of these ECM proteins, especially the ratio of elastin to collagen, determines arterial mechanical properties and affects SMC morphology and function.^61^ Several studies have reported that after removal of elastin, collagen fibers are no longer constrained and become disordered,^63^ and structural changes in adventitial collagen associated with elastin degradation include reduced waviness,^47^ leading to an elevated stiffness and reduced extensibility.^63^

Due to collagen's intrinsic properties such as optical anisotropy, fibril structure, and multifibril organization, collagen fibers and bundles were visualized in this study without labeling agents under SHG with femtosecond pulsed infrared excitation.^66^ The SHG technique offers many advantages for biomedical assessment of tissue structure, including visualization of collagen fibers without staining and deeper penetration within a sample when compared with conventional single‐photon excitation microscopy.^34^ SHG emission directionality is sensitive to the diameter of the fibrils that are bundled into collagen fibers, as well as their spacing within the fiber, and the disorder in their packing.^67^, ^68^, ^69^ Mature fibrils produce a more prominent forward channel signal, whereas immature fibril segments produce relatively more backward‐directed SHG.^69^ The ratio of the forward emitted to backward‐emitted SHG (the F/B ratio) is thus sensitive to differences in the structural properties of collagen fibers.^35^

We found a significant increase in F/B ratio in skeletonized vessels compared to controls, indicating a difference in collagen structure. Although the details of these collagen differences were not directly assessed in this study, collagen maturation is typified by qualitative and quantitative alterations in crosslinking. Changes in collagen content and organization are mediated by the adventitial fibroblasts, which are activated is response to stress or injury. The skeletonization of the vessel likely results in the activation of adventitial fibroblasts, leading to a change in ECM production and organization, especially medial elastin, which have profound effects on vessel function.^70^ Interestingly, alterations in collagen play a significant role in vessel stiffening associated with the development of aneurysms, atherosclerosis, and hypertension.^34^, ^51^, ^62^ The changes in both elastin and collagen organization noted in our study suggest that the decrease in cyclic strain in skeletonized vessels is due to either a reduction in elastic recoil during diastole associated with elastin disruption, reduced distension during systole associated with alterations in collagen, or both.

Based on their known wound‐healing properties,^42^, ^43^ we hypothesized that a PEG‐based hydrogel would protect the skeletonized vessel from the observe tissue damage and alterations in cyclic strain. We found that the addition of hydrogel abrogated the effects seen with surgical skeletonization alone. Circumferential cyclic strain, collagen organization, and the number of elastic laminae of vessels receiving hydrogel were all similar to unoperated control vessels. While hydrogel treated vessels had an immediate drop in cyclic strain, this may result from the equilibration of its fluid balance and increased local edema, as the cyclic strain returned to the baseline level over the course of the study. Overall, the application of 4 wt% PEG hydrogel immediately after surgical manipulation resulted in dramatic improvements in the operated vessel compared to skeletonized vessels that did not receive hydrogel.

Although detailed studies of the fate of injected hydrogels were outside the scope of the current work, intact hydrogel was visible around the artery immediately after injection and by ultrasonography through the 21‐day time course of the experiments (data not shown). Although the hydrogels were not engineered for hydrolytic or enzymatic degradation, we expect that the hydrogels likely experienced some degradation over time via mechanical abrasion or PEG chain scission by local reactive oxygen intermediates.^71^ As it may be desirable for hydrogels to degrade over time, future engineering of hydrogels with controlled degradation profiles is anticipated. Future studies will investigate the fate of implanted hydrogels, as well as the underlying mechanism by which hydrogels protect against tissue damage caused by skeletonization. We suspect that the hydrogel may act as a lubricating layer between the injured surface of the vessel and its surroundings and that it may also act to block recruitment of inflammatory cells to the site of the injury. Interestingly, one such cell type, the rabbit heterophil, contains large amounts of elastase activity and is known to be rapidly recruited to sites of injury.^72^

**Figure 6:**
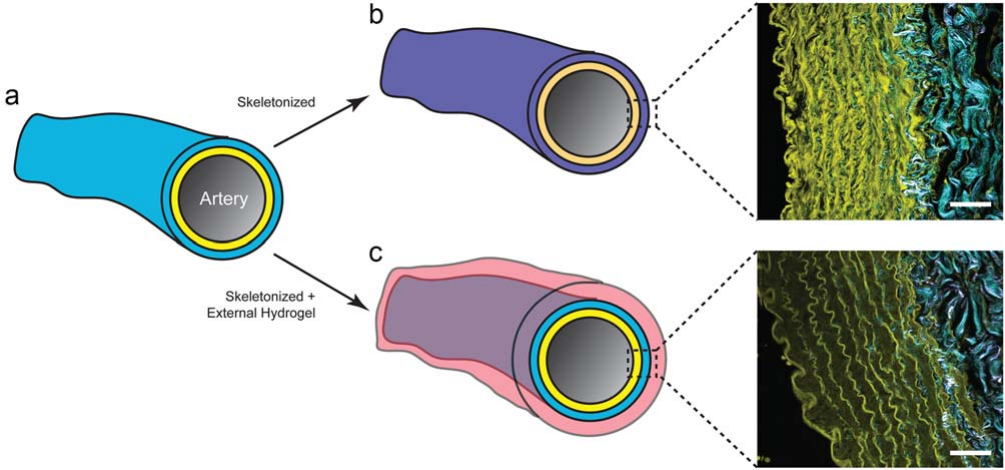
(a) Surgical artery grafting and reconstruction are principal treatments for coronary artery disease, peripheral artery disease, and congenital cardiovascular malformations. (b) Arteries may be skeletonized for harvesting of graft tissue. Within 21 days of surgery, skeletonized arteries exhibit reduced circumferential cyclic strain, fragmented medial elastin (visualized by TPEF, yellow), and alterations in adventitial collagen turnover (visualized by SHG, cyan, and magenta). (c) PEG hydrogel (pink) injected around the abluminal surface of the artery at the time of skeletonization ameliorates these effects and may be used as an adjunctive approach to surgical manipulations involving blood arteries. Scale bars = 25 μm

Our results suggest that simple, injectable gels may be useful as an adjunctive approach to critical surgical manipulations involving arterial skeletonization (Figure 6), and that hydrogels may be especially beneficial during the acute inflammatory phase of postsurgical recovery. To the authors' knowledge, current surgical standard of care does not include the placement of any materials on the abluminal surface of skeletonized blood vessels. Strategies for wrapping anastomotic sites^73^ and for the use of peri‐adventitial materials for drug delivery^74^ have been described in the research literature; however, these have not yet been adopted for clinical use. Given their simplicity, ease of delivery, and optimizability, the hydrogel materials described in the present work may have clinical applications for vascular grafting, congenital cardiovascular repair, arterial skeletonization during transplant or reconstructive surgery, maintenance of vascular access, and surgical procedures involving dissection of arterial tissues. In addition, the hydrogel system could be devised with a multiplicity of formulations to drive desired cellular responses, including alterations in polymer weight percent to match desired physical characteristics, the addition of anti‐inflammatory or immunosuppresive agents, or the incorporation of stem cells to further enhance postsurgical recovery.
